# Prevalence and prediction of masked uncontrolled hypertension in patients recently hospitalized for myocardial infarction

**DOI:** 10.1093/ehjopen/oeaf138

**Published:** 2025-10-14

**Authors:** Henrik Hellqvist, David Erlinge, Bertil Lindahl, Tomas Jernberg, Jonas Oldgren, Stefan James, Faris Al-Khalili, Thomas Kahan, Jonas Spaak

**Affiliations:** Division of Cardiovascular Medicine, Department of Clinical Sciences, Danderyd Hospital, Karolinska Institutet, SE-182 88 Stockholm, Sweden; Department of Clinical Sciences, Lund, Cardiology, Lund University, BMC I12, SE-221 84 Lund, Sweden; Department of Medical Sciences, Cardiology, Uppsala University, Akademiska sjukhuset, SE-751 85 Uppsala, Sweden; Uppsala Clinical Research Center, Uppsala University, Uppsala Science Park, Dag Hammarskjölds väg 38, SE-751 85 Uppsala, Sweden; Division of Cardiovascular Medicine, Department of Clinical Sciences, Danderyd Hospital, Karolinska Institutet, SE-182 88 Stockholm, Sweden; Department of Medical Sciences, Cardiology, Uppsala University, Akademiska sjukhuset, SE-751 85 Uppsala, Sweden; Uppsala Clinical Research Center, Uppsala University, Uppsala Science Park, Dag Hammarskjölds väg 38, SE-751 85 Uppsala, Sweden; Department of Medical Sciences, Cardiology, Uppsala University, Akademiska sjukhuset, SE-751 85 Uppsala, Sweden; Uppsala Clinical Research Center, Uppsala University, Uppsala Science Park, Dag Hammarskjölds väg 38, SE-751 85 Uppsala, Sweden; Division of Cardiovascular Medicine, Department of Clinical Sciences, Danderyd Hospital, Karolinska Institutet, SE-182 88 Stockholm, Sweden; Division of Cardiovascular Medicine, Department of Clinical Sciences, Danderyd Hospital, Karolinska Institutet, SE-182 88 Stockholm, Sweden; Division of Cardiovascular Medicine, Department of Clinical Sciences, Danderyd Hospital, Karolinska Institutet, SE-182 88 Stockholm, Sweden

**Keywords:** Masked hypertension, Machine learning, Myocardial infarction

## Abstract

**Aims:**

To study the prevalence of masked uncontrolled hypertension (MUCH) in patients recently hospitalized for myocardial infarction, and to develop machine learning-based prediction models identifying MUCH.

**Methods and results:**

Ambulatory blood pressure measurement (ABPM) was performed in 99 patients following hospitalization for a myocardial infarction. Sixty-two clinical variables were eligible for machine learning. Variable importance for the prediction of MUCH (office blood pressure <140/90 mm Hg at ABPM start but mean 24-h blood pressure ≥130/80 mm Hg) was assessed using the least absolute shrinkage and selection operator (LASSO) and the Boruta algorithms. Logistic regression, LASSO, and random forest models based on the top variables were evaluated using receiver operating characteristic area under the curve (AUC) in repeated cross-validation. Mean age was 62.1 ± 8.2 years, 73 (74%) were males. The ABPM was performed at a median of 11 weeks after discharge. Among 96 patients with valid 24-h ABPM recordings, 32 (33%) had 24-h mean blood pressure ≥130/80 mm Hg and 17 (18%) were identified with MUCH. Machine learning identified discharge diagnoses of diabetes and hypertension, and kidney dysfunction as most important predictors of MUCH. The best random forest, logistic regression, and LASSO models showed mean AUC 0.82, 0.80, and 0.80, respectively, for prediction of MUCH.

**Conclusion:**

One in five patients had MUCH at follow-up after a myocardial infarction. The readily available variables diabetes, hypertension, and kidney dysfunction were identified as the most important predictors of MUCH, which may be implemented in a prediction model for identifying this clinically challenging blood pressure phenotype.

**Previous presentation:**

Preliminary results were presented at the European Society of Cardiology Congress in London 2024 as an oral abstract presentation. Hellqvist H, Erlinge D, Lindahl B*, et al.* Prevalence and prediction of masked uncontrolled hypertension in patients recently hospitalised for an acute coronary syndrome. *European Heart Journal* 2024;45 (Suppl 1). doi: 10.1093/eurheartj/ehae666.2566

## Graphical Abstract

**Graphical Abstract:**
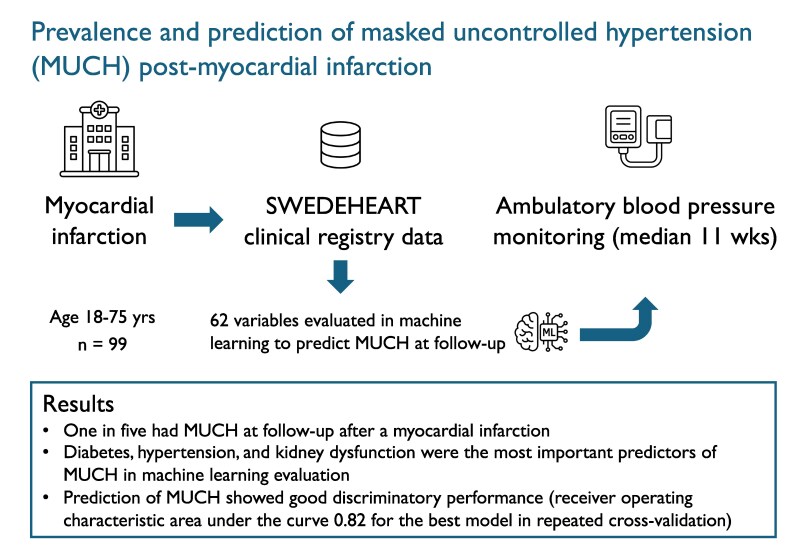

## Introduction

After an acute myocardial infarction (MI), patients face a sustained elevated risk for new cardiovascular events, which makes secondary prevention strategies including healthy lifestyle, and risk factor control important.^1,2^ Hypertension is a major cardiovascular risk factor,^3,4^ and studies have shown that lowering blood pressure is equally important for both primary and secondary prevention of future cardiovascular events.^5^ However, hypertension remains common in the setting of secondary prevention after a MI.^3,6^

Four blood pressure phenotypes can be identified by recording office blood pressure and out-of-office blood pressure (assessed by home or ambulatory blood pressure monitoring, ABPM). These phenotypes include normotension (neither office blood pressure nor out-of-office blood pressure is elevated), white-coat hypertension (WCH; elevated office blood pressure but not out-of-office blood pressure), sustained hypertension (both office blood pressure and out-of-office blood pressure elevated), and masked hypertension (normal office blood pressure but elevated out-of-office blood pressure). Patients who remain hypertensive while on antihypertensive treatment are referred to as uncontrolled, which defines the sub-phenotypes sustained uncontrolled hypertension, white-coat uncontrolled hypertension and masked uncontrolled hypertension (MUCH).^3,7^

Masked hypertension presents a clinical challenge, as it remains undetected without out-of-office blood pressure measurements.^3,7,8^ Masked hypertension, untreated or treated, affects 10–20% of patients attending hypertension clinics,^7^ whereas a higher prevalence has been reported for patients with specific comorbidities like obstructive sleep apnoea (OSA; up to 30%), diabetes (13–66%) and chronic kidney disease (7–33%).^8^ Masked hypertension has been shown to often progress to sustained hypertension,^9^ and carries a cardiovascular risk similar to that of sustained hypertension, both in the general population,^8,10^ and in patients with diabetes or chronic kidney disease.^11^

Since feasibility and costs limit screening of all patients with out-of-office blood pressure measurements, some studies have attempted to develop prediction models as screening tools for masked hypertension.^12–14^ However, although promising, machine learning approaches for the evaluation of hypertension remain under-used.^15^ In addition, these previous studies have primarily been performed in patients with hypertension and not in patients with established cardiovascular disease. Given the further increased risk caused by poorly controlled blood pressure in patients with MI,^16^ the primary aim of this study was to investigate the prevalence of masked hypertension and MUCH in patients following a recent MI. The secondary aim was to develop a machine learning method to predict masked hypertension and MUCH from structured clinical registry data.

## Methods

### Study design and participants

**Figure 1:**
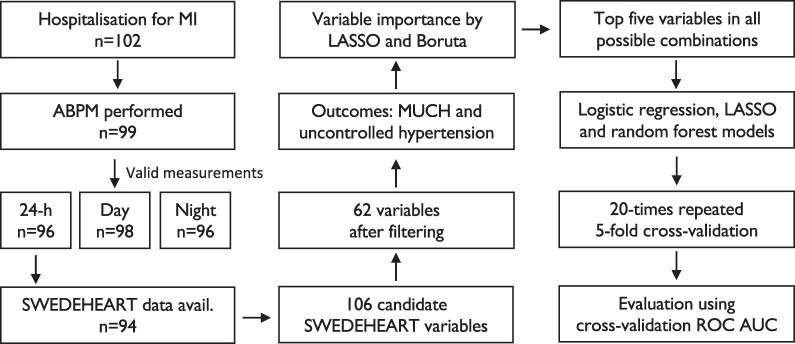
Study flowchart. MI, myocardial infarction; ABPM, ambulatory blood pressure monitoring; SWEDEHEART, a Swedish nationwide quality registry covering acute coronary syndromes; LASSO, least absolute shrinkage and selection operator; MUCH, masked uncontrolled hypertension; ROC AUC, receiver operating characteristic area under the curve.

In 2016–2018, 102 patients aged 18–75 years, and hospitalized for an acute MI, at the Department of Cardiology, Danderyd University Hospital, Stockholm (Sweden), were included during out-patient follow-up, provided they had no terminal disease with life expectancy less than two years and no known ongoing or recent bleeding complications. The study was approved by the regional ethic review board (2016/1302-31), Stockholm (Sweden), conducted in accordance with the Declaration of Helsinki, and written informed consent was obtained from each subject prior to study participation. The first physician follow-up, ABPM, and second physician follow-up took place at 7 [7–8], 11 [9–15] and 54 [53–56] weeks after hospital discharge, respectively. In patients with hypertension by ABPM the investigators either intensified the antihypertensive medications and/or sent a referral to their treating physician. A study flowchart is presented in *Figure 1*.

### Assessment of clinical characteristics

Data on clinical characteristics were almost exclusively obtained from the SWEDEHEART (Swedish Web-system for Enhancement and Development of Evidence-based care in Heart disease Evaluated According to Recommended Therapies) registry.^17^ This registry contains nationwide coverage on Swedish patients who have been hospitalized for an acute coronary syndrome, and their structured follow-up. The registry encompasses more than 200 clinical variables (including comorbidities, routine laboratory tests, examinations, and treatments) from the index hospital stay, the first and the second out-patient physician follow-up at 6–10 weeks and 12–14 months, respectively.

### Blood pressure measurements defining blood pressure phenotypes

An office blood pressure was measured in the seated position with the auscultatory method, using a proper cuff size, in both arms, at the initiation of the ABPM. If there was an inter-arm blood pressure difference, the arm with the highest blood pressure was used for office blood pressure and ABPM, if not, according to patient preference. ABPM was performed using Novacor Diasys Integra 2 (Novacor, Rueil-Malmaison, France) on the upper arm with recordings every 20 min throughout. The recordings were processed in the HolterSoft Ultima version 2.4.4 software (Novacor, Rueil-Malmaison, France). The subject-reported bedtime and wake-up times were used to define the periods being awake and asleep. ABPM recordings were considered valid if they included at least 20 valid awake and 7 valid asleep measurements. In five patients with 12–19 awake recordings, the awake period was manually reviewed and marked as valid based on acceptable variability and spread of values during the period.

### Blood pressure phenotypes definitions

A 24-h, day-time and night-time hypertension by ABPM was defined as a mean (systolic and/or diastolic) blood pressure ≥130/80, ≥135/85, and ≥120/70 mm Hg, respectively, and office blood pressure hypertension was defined as ≥140/90 mm Hg.^4,7^ Blood pressure phenotypes were defined by using the office blood pressure at the time of the initiation of the ABPM equipment, giving the following phenotypes: normotension, WCH, sustained hypertension and masked hypertension (the two latter were defined as uncontrolled if the patient was on antihypertensive medication).^7^ Antihypertensive medications were the ones reported at the time of ABPM and if this information was missing, medications from the first follow-up (*n* = 7) or discharge (*n* = 1) were used as proxy. Uncontrolled hypertension was defined as the combination of sustained uncontrolled hypertension and MUCH.^7^ Unless stated otherwise, the 24-h blood pressure levels were used for the definitions of blood pressure phenotypes. This definition was chosen because 24-h blood pressure data is more robust, includes night-time hypertension, which has a stronger prognostic value than day-time hypertension, and has recently attracted more attention in the definition of masked hypertension.^4,8^ Dipping was defined as a reduction in mean awake-to-asleep systolic and/or diastolic blood pressure of ≥10%. Office pulse pressure was calculated as systolic minus diastolic blood pressure.

### Statistical analysis and machine learning

Descriptive data are presented as mean values ± standard deviation, median values with interquartile ranges, and proportions, as appropriate. Comparisons between groups for continuous variables were assessed by one-way analysis of variance or by Kruskal–Wallis test, as appropriate, and for categorical variables by the Chi-squared test or, when frequencies were below five, by Fisher’s exact test. Testing between-group differences were corrected for multiple comparisons using the Bonferroni method. A two-sided probability level (*P*) < 0.05 was taken as significant.

For machine learning and the development of prediction models, a selection of variables representing easily accessible clinical patient characteristics from the hospitalization period and the first follow-up visit was made. This resulted in 106 candidate variables that were available for further processing (see Supplementary material online, *Table S1*). These variables were pre-processed by filtering on over six missing values (missing values were imputed using the k-nearest neighbour algorithm),^18^ zero or near-zero variance, and for logistic regression and least absolute shrinkage and selection operator (LASSO)^19^ algorithms, also by centering, scaling and Box-Cox transformation to improve normality. Variable importance for the prediction of MUCH was assessed using LASSO and Boruta^20^ machine learning algorithms. The Boruta algorithm relies on random forest variable importance scores and selects variables that outperform randomized copies created from the original predictors, while LASSO uses a predictor coefficient penalty leading to removal of unimportant predictors. The top five variables identified by LASSO and by Boruta, respectively, were combined into one variable group for each of the outcomes, MUCH and uncontrolled hypertension. Subsequently, logistic regression, LASSO, and random forest models, using all possible combinations within each variable group, were evaluated by receiver operating characteristic area under the curve (ROC AUC) in five-fold cross-validation with stratification of the outcome, repeated 20 times, as the resampling method. The mean predicted probability for each observation was used to construct ROC curves, and the threshold at maximum Youden’s index was used to calculate sensitivity, specificity, positive predictive value, negative predictive value, and accuracy. A study flowchart including machine learning is presented in *Figure 1*.

Statistical analyses and machine learning were performed using the software R (version 4.4.1; R Foundation for Statistical Computing, Vienna, Austria). Machine learning-specific R packages included Boruta (version 8.0.0), glmnet (version 4.1.8) for logistic regression and LASSO modelling, and ranger (version 0.16.0) for random forest modelling. The latter two were integrated using the tidymodels framework (version 1.2.0).

## Results

### Clinical characteristics according to blood pressure phenotype

In total 102 patients were recruited, out of which 99 underwent ABPM (*Figure 1*). *Table 1* presents the study population according to blood pressure phenotype. The majority were male, approximately half were current or previous smokers, and STEMI (ST-elevation MI) and NSTEMI (non-ST-elevation MI) were equally common. All participants with hypertension by ABPM were on at least one medication with antihypertensive properties at the time of ABPM, and as such considered uncontrolled, having either sustained uncontrolled hypertension or MUCH.

**Table 1:** Clinical characteristics according to 24-h blood pressure phenotype post-myocardial infarction

Characteristic	All	Normotension	White-coat hypertension	Masked hypertension	Sustained hypertension	*P*	*Q*
*n*	99	59	5	17	15		
Age, years	62.1 ± 8.2	61 ± 8	65 ± 13	65 ± 9	62 ± 8	0.046	>0.99
Male sex	73 (74%)	41 (69%)	4 (80%)	15 (88%)	10 (67%)	0.45	>0.99
Previous MI	7 (7.2%)	5 (8.6%)	0 (0%)	0 (0%)	0 (0%)	0.63	>0.99
BMI, kg/m^2^	27.8 ± 4.3	27.4 ± 4.0	32.0 ± 5.1	27.6 ± 4.1	27.6 ± 5.2	0.15	>0.99
Current or previous smoking	46 (48%)	25 (43%)	2 (40%)	8 (47%)	9 (64%)	0.57	>0.99
LDL, mmol/L	3.1 ± 1.1	3.13 ± 1.13	2.39 ± 0.99	3.05 ± 1.09	3.51 ± 0.80	0.27	>0.99
eGFR, mL/min/1.73m^2^	89 ± 15	90 ± 15	95 ± 10	80 ± 14	94 ± 15	0.034	0.86
HbA1c, mmol/L	38 [35;42]	37 [35;41]	36 [34;39]	41 [37;47]	41 [38;44]	0.043	>0.99
Max troponin T, mmol/L	402 [98;1170]	417 [97;1170]	475 [144;7350]	178 [107;1000]	314 [79;1270]	0.93	>0.99
LVEF ≥50%	65 (72%)	39 (71%)	3 (60%)	11 (73%)	10 (83%)	0.76	>0.99
MI type (NSTEMI/STEMI)						0.34	>0.99
NSTEMI	50 (52%)	27 (47%)	2 (40%)	12 (71%)	7 (50%)		
STEMI	47 (48%)	31 (53%)	3 (60%)	5 (29%)	7 (50%)		
AF/AFL diagnosis	9 (9.3%)	5 (8.6%)	0 (0%)	2 (12%)	1 (7.1%)	0.91	>0.99
COPD diagnosis	1 (1.0%)	0 (0%)	0 (0%)	0 (0%)	1 (7.1%)	0.20	>0.99
OSA diagnosis	4 (4.1%)	1 (1.7%)	1 (20%)	2 (12%)	0 (0%)	0.072	>0.99
Hypertension diagnosis	58 (60%)	28 (48%)	4 (80%)	15 (88%)	9 (64%)	0.014	0.36
Heart failure diagnosis	17 (18%)	11 (19%)	0 (0%)	4 (24%)	2 (14%)	0.81	>0.99
Diabetes diagnosis	19 (20%)	7 (12%)	0 (0%)	7 (41%)	3 (21%)	0.038	0.94
Impaired OGTT diagnosis	20 (21%)	14 (24%)	0 (0%)	1 (5.9%)	4 (29%)	0.23	>0.99
BP-lowering medication at ABPM	99 (100%)	59 (100%)	5 (100%)	17 (100%)	15 (100%)		
ACE-inhibitor	59 (60%)	36 (61%)	3 (60%)	9 (53%)	9 (60%)	0.94	>0.99
ARB	29 (29%)	14 (24%)	2 (40%)	7 (41%)	5 (33%)	0.42	>0.99
Beta-blocker	80 (81%)	51 (86%)	3 (60%)	14 (82%)	9 (60%)	0.072	>0.99
CCB	15 (15%)	8 (14%)	1 (20%)	3 (18%)	2 (13%)	0.89	>0.99
MRA	3 (3.0%)	3 (5.1%)	0 (0%)	0 (0%)	0 (0%)	>0.99	>0.99
Thiazid diuretic	3 (3.0%)	1 (1.7%)	1 (20%)	0 (0%)	0 (0%)	0.21	>0.99
Alfa-blocker	0 (0%)	0 (0%)	0 (0%)	0 (0%)	0 (0%)	>0.99	>0.99

Values presented as mean values ± standard deviation, median values [interquartile range], or *n* (%), with *P* level of significance between-groups and *Q*, showing adjusted *P* values after correction for multiple testing. Of the 99 patients with ambulatory blood pressure measurements (ABPM), 96 were valid for 24-h blood pressure phenotype classification. Clinical characteristics were present at hospital discharge. Blood pressure (BP) medications are the ones reported at the time of ABPM. MI, myocardial infarction; BMI, body mass index; LDL, low-density lipoprotein cholesterol; eGFR, estimated glomerular filtration rate; LVEF, left ventricular ejection fraction; STEMI, ST-elevation MI; NSTEMI, non-ST-elevation MI; AF/AFL, atrial fibrillation/flutter; COPD, chronic obstructive pulmonary disease; OSA, obstructive sleep apnoea; OGTT, oral glucose tolerance test; ACE, angiotensin-converting enzyme; ARB, angiotensin II receptor blocker; CCB, calcium channel blocker; MRA, mineralocorticoid receptor antagonist.

### Office blood pressure at hospitalization and follow-up

**Figure 2:**
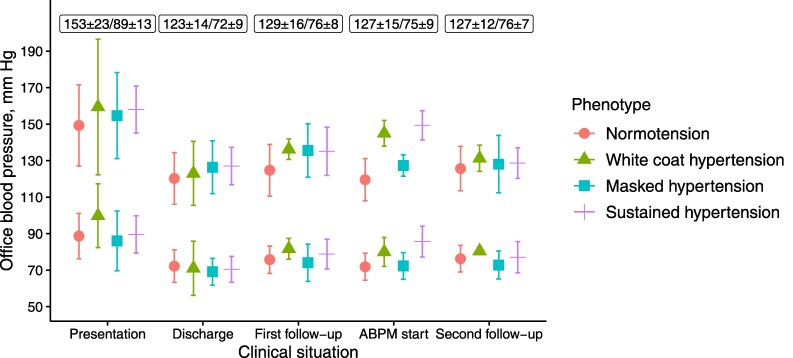
Systolic and diastolic blood pressure levels at different clinical situations according to blood pressure phenotype. Central symbols represent the means, and vertical lines the standard deviations (SD). Each box contains the total mean systolic and diastolic blood pressure values ± SD for respective situation. The first follow-up, ambulatory blood pressure monitoring (ABPM) and second follow-up took place at 7 [7–8], 11 [9–15], and 54 [53–56] weeks, respectively, after hospital discharge.

Office blood pressure levels during the hospitalization were higher at presentation and lower at discharge, as expected (*Figure 2*). Office blood pressure levels were comparable at first follow-up, ABPM start, and at second follow-up (*Figure 2*). At presentation, discharge, first follow-up, ABPM start, and second follow-up, 18, 83, 72, 80, and 91%, had a systolic and diastolic blood pressure <140 and 90 mm Hg, (i.e. no hypertension), respectively. The spread of office blood pressure values among the different blood pressure phenotypes was more visible at first follow-up and at ABPM start (*Figure 2*). Out of the 17 (18%) participants with MUCH, 10 (63%) had no hypertension at first follow-up and 8 (53%) and no hypertension at both discharge, first follow-up and ABPM start.

### Ambulatory blood pressure monitoring during follow-up

**Figure 3:**
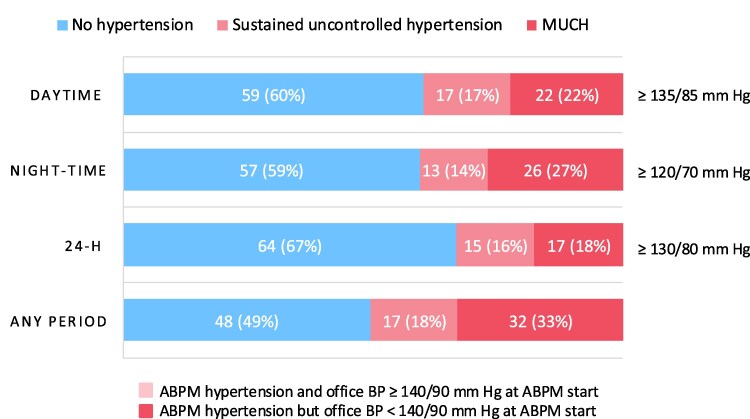
Proportions of patients without hypertension, with sustained uncontrolled hypertension and masked uncontrolled hypertension (MUCH) post-myocardial infarction, as detected by ambulatory blood pressure monitoring (ABPM). Percentages are rounded to whole numbers. BP, blood pressure.

Of all 102 patients, 99 underwent ABPM, and 96, 98, and 96 provided valid recordings for the 24-h, day and night periods, respectively (*Figure 1*). Uncontrolled hypertension was prevalent, affecting 33% of the participants for the 24-h period and 51% for any period; and 53–67% of the participants with uncontrolled hypertension had MUCH across the different periods (*Figure 3*). ABPM metrics according to blood pressure phenotype are presented in Supplementary material online, *Table S2*.

### Prediction of MUCH

Among 96 patients with valid 24-h periods on ABPM, two had missing registry data, resulting in 94 patients who were used in machine learning for prediction of MUCH and uncontrolled hypertension (*Figure 1*). In all, 106 candidate variables underwent filtering on more than six missing values (11 variables were removed, and 36 of the remaining variables had on average 3.5 values imputed) and low variance (33 variables removed), which resulted in 62 variables which were finally eligible for machine learning (see Supplementary material online, *Table S1*).

**Figure 4:**
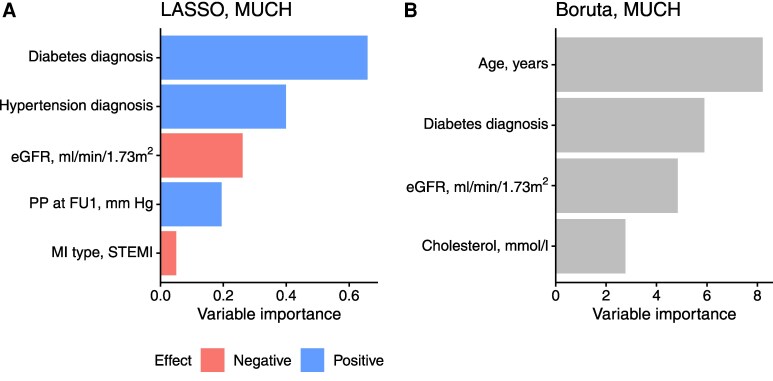
Variable importance scores from machine learning in prediction of masked uncontrolled hypertension (MUCH) using (*A*) LASSO and (*B*) Boruta algorithms. The scores use relative scales, and LASSO also includes the effect direction. Myocardial infarction (MI) type refers to STEMI (ST-elevation MI) or NSTEMI (non-ST-elevation MI). LASSO, least absolute shrinkage and selection operator; eGFR, estimated glomerular filtration rate; PP, pulse pressure; FU1, first follow-up.

Discharge diagnoses of diabetes and hypertension, estimated glomerular filtration rate (eGFR), pulse pressure at first follow-up, MI type (STEMI or NSTEMI), age, and cholesterol levels were found to be the most important predictors of MUCH (*Figure 4 A-B*, Supplementary material online, *Table S3*). All combinations of these variables resulted in 127 sets of predictors that subsequently were evaluated in machine learning.

**Figure 5:**
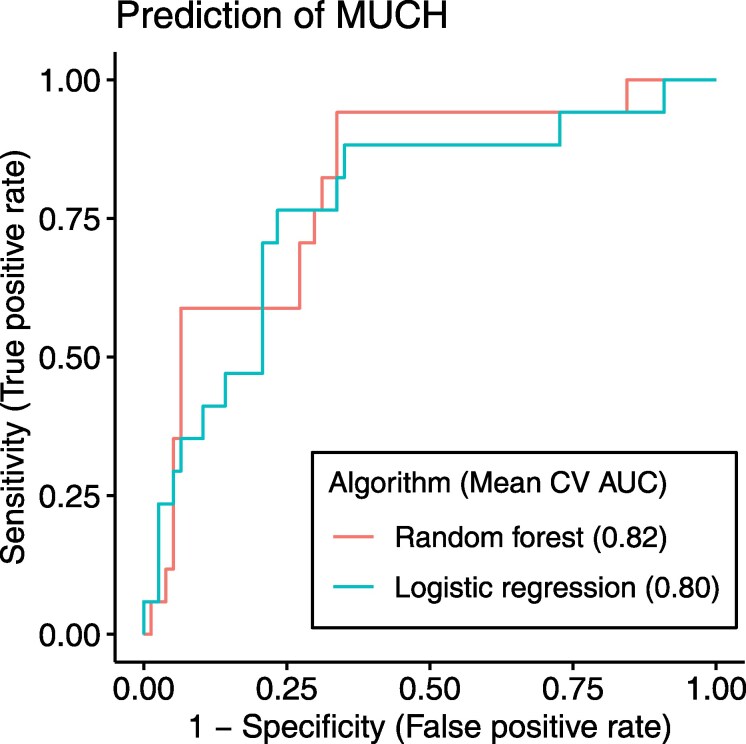
Receiver operating characteristic (ROC) curves constructed using the mean probability from repeated cross-validation (CV) for each observation, showing the best random forest and logistic regression models with each mean CV area under the curve (AUC) value presented. The best least absolute shrinkage and selection operator (LASSO) model had a similar ROC curve and AUC value as the best logistic regression model, and is not shown in the figure.

Prediction of MUCH is presented in *Table 2*. The highest mean cross-validation AUC was seen for a random forest model using discharge diagnoses of diabetes and hypertension, eGFR, and age as predictors (*Table 2*, *Figure 5*). Comparable discriminative performance was observed for a logistic regression model using diabetes, hypertension and eGFR as predictors (*Table 2*, *Figure 5*), variables which overall were included in most models with the highest mean cross-validation AUC. The model with the highest mean cross-validation AUC provided a sensitivity of 0.94, specificity of 0.66, positive predictive value of 0.38, negative predictive value of 0.98, and an accuracy of 0.71 (*Table 2*).

**Table 2:** Prediction of masked uncontrolled hypertension post-myocardial infarction

Algorithm	Predictors	Mean CV AUC	Sensitivity	Specificity	PPV	NPV	Accuracy
Random forest	Diabetes; Hypertension; eGFR; Age	0.82	0.94	0.66	0.38	0.98	0.71
Random forest	Diabetes; eGFR; Age	0.81	0.94	0.57	0.33	0.98	0.64
Random forest	Diabetes; Hypertension; eGFR; Age; Cholesterol	0.81	0.88	0.69	0.38	0.96	0.72
Random forest	Diabetes; eGFR; Age; Cholesterol	0.81	0.65	0.88	0.55	0.92	0.84
Random forest	Diabetes; Hypertension; eGFR; MI type; Age	0.80	0.76	0.73	0.38	0.93	0.73
Logistic regression	Diabetes; Hypertension; eGFR	0.80	0.88	0.65	0.36	0.96	0.69
LASSO	Diabetes; Hypertension; eGFR	0.80	0.88	0.65	0.36	0.96	0.69
LASSO	Diabetes; Hypertension; eGFR; MI type	0.79	0.71	0.77	0.40	0.92	0.76
Logistic regression	Diabetes; Hypertension; eGFR; MI type	0.79	0.71	0.77	0.40	0.92	0.76
LASSO	Diabetes; eGFR	0.79	0.82	0.68	0.36	0.95	0.70
Logistic regression	Diabetes; eGFR	0.79	0.82	0.68	0.36	0.95	0.70
Logistic regression	Diabetes; Hypertension; eGFR; Cholesterol	0.79	0.76	0.73	0.38	0.93	0.73
LASSO	Diabetes; Hypertension; eGFR; Cholesterol	0.78	0.76	0.73	0.38	0.93	0.73
LASSO	Diabetes; eGFR; PP at FU1	0.78	0.71	0.77	0.40	0.92	0.76
Logistic regression	Diabetes; eGFR; PP at FU1	0.78	0.71	0.77	0.40	0.92	0.76

Prediction of masked uncontrolled hypertension using different machine learning algorithms in an exhaustive evaluation of all top predictors from variable importance analyses. The list is sorted on mean repeated cross-validation (CV) receiver operating characteristic area under the curve (AUC). The mean predicted probability for each observation and the threshold at maximum Youden's index were used to calculate the performance metrics sensitivity, specificity, positive predictive value (PPV), negative predictive value (NPV) and accuracy. Diabetes and hypertension represent diagnoses at discharge. eGFR, estimated glomerular filtration rate; MI, myocardial infarction; LASSO, least absolute shrinkage and selection operator; PP, pulse pressure; FU1, first follow-up.

### Prediction of uncontrolled hypertension

Discharge diagnoses of diabetes and hypertension, and level of physical activity, systolic blood pressure, and pulse pressure at first follow-up were the top predictors of uncontrolled hypertension (see Supplementary material online, *Figure S1 A*-*B*, Supplementary material online, *Table S3*), resulting in 31 sets of predictors that subsequently were evaluated in machine learning. The highest mean cross-validation AUC was seen for a random forest model using discharge diagnosis of diabetes, and level of physical activity, systolic blood pressure, and pulse pressure at first follow-up as predictors (see Supplementary material online, *Table S4*).

### Discussion

This appears to be the first study to report on the prevalence of MUCH, and the prediction of MUCH by applying machine learning methods in subjects at very high cardiovascular risk, i.e. a recent hospitalization for an acute MI. This study provides four main findings. First, MUCH is common in patients with a recent MI. Second, MUCH in these patients can be predicted by machine learning models with good discriminatory performance. Third, the predictors in this group of very high-risk patients are different from those reported in populations with lower cardiovascular risk. Fourth, uncontrolled hypertension following a MI was highly prevalent and may also be identified through machine learning models.

#### Prevalence of uncontrolled hypertension and MUCH

The prevalence of uncontrolled hypertension overall, following a MI, was high, affecting up to half of the patients considering any period (day, night or 24-h). The prevalence of MUCH in our study is on par with findings in international registries that use the same definition of MUCH.^21^ The proportion of patients that had an office blood pressure <140/90 mm Hg, i.e. no hypertension, at second follow-up was high (91%), which may be because we intervened in patients with hypertension on ABPM.

#### Prediction of MUCH

We performed an exhaustive machine learning-based development of prediction models. The best model predicting MUCH provided an AUC of 0.82, where an AUC of >0.8 generally is considered to offer excellent discriminatory performance,^22^ a high sensitivity (0.94) and a high negative predictive value (0.98) which is favourable for prediction of a condition like MUCH.

Our results show comparable AUC values to a few other studies on the prediction of masked hypertension/MUCH, with a similar approach to ours, but in different populations and with a smaller number of clinical variables. These include a study by Kim et al that developed prediction models in 1986 South Korean individuals (13% previous MI) treated for hypertension (AUC 0.84 in internal validation),^13^ a study by Hung et al in based on 970 individuals in a Taiwanese hypertensive population with no diabetes and no medical history of severe diseases, aged 20–50 years (AUC 0.80–0.85 in internal validation, 0.67–0.84 in external validation),^14^ and a study by Coccina et al based on 738 treated individuals in a hypertensive Italian population (AUC 0.77–0.87 in internal validation).^12^

#### Predictors of MUCH

For variable selection we applied both LASSO and Boruta. That these do not provide the exact same results can be explained by the different approaches of the algorithms, which justify using both. LASSO is based on penalized regression, which identifies predictors with linear associations with the outcome,^19^ whereas Boruta relies on random forest variable importance and can capture non-linear relationships and interactions between predictors.^20,23^ In addition, the Boruta algorithm compares variables with randomized ‘shadow’ copies of the original ones, which provides a built-in statistical analysis for variable selection.

The discharge diagnoses diabetes and hypertension, eGFR, and age were the most important predictors of MUCH, forming the model with the highest mean cross-validation AUC. However, models containing only diabetes, hypertension and eGFR performed similarly well, suggesting that the predictive ability using only these predictors is sufficient. All these predictors are readily available during the hospital stay, which facilitates a prediction even before the patient is discharged from the hospital.

The fact that diabetes was important for the prediction of MUCH falls in line with previous studies showing diabetes to be associated with masked hypertension,^24,25^ and diabetes to be more prevalent among MUCH patients compared to those with controlled hypertension.^26^ Diabetes leads to increased arterial stiffness through vascular remodelling,^27^ which previously has been linked to masked hypertension.^28,29^ Increased arterial stiffness impairs blood pressure buffering, leading to greater blood pressure variability, which reduces the accuracy of sporadic office blood pressure measurements in assessing hypertension and blood pressure control. Supporting this notion, pulse pressure, an easily acquired indirect measure of arterial stiffness,^3^ was also found to be an important predictor of MUCH in our study.

Kidney dysfunction (eGFR) was also an important predictor of MUCH in our study, and has previously been associated with masked hypertension and MUCH.^25,30,31^ The pathophysiological explanations include a tendency for nocturnal hypertension,^31^ which goes undetected by office blood pressure, and through increased arterial stiffness due to accelerated vascular ageing and vascular calcification.^28^

Of note, both diabetes and kidney dysfunction cause increased sympathetic activity,^26,32^ which contributes to an exaggerated blood pressure response to stress and physical activity, which even further challenges the use of office blood pressure measurements for blood pressure assessment in these patients.

A history of hypertension has previously been linked to masked hypertension/MUCH.^25^ This may be because office blood pressure, normally recorded during day-time working hours, often occur during the peak of the antihypertensive medication effects,^8^ and does not reveal elevated evening or night-time blood pressure which may contribute to a masked hypertension/MUCH phenotype. Taken together, the individual components of our best prediction models for MUCH align well with previously reported markers associated with masked hypertension/MUCH.

Compared to the above-mentioned studies on the prediction of masked hypertension/MUCH we identified other results of interest regarding importance and selection of variables. The study by Kim et al evaluated 19 clinical characteristics, resulting in a logistic regression model including office systolic and diastolic blood pressure, stroke, dyslipidaemia, left ventricular hypertrophy, heart rate, and number of antihypertensive drugs.^13^ Hung et al evaluated 33 characteristics and found best predictive performance in models using office systolic, diastolic and mean arterial blood pressure, pulse pressure, beta-blocker use, and high density lipoprotein cholesterol as predictors.^14^ Furthermore, Coccina et al found that male sex, smoking, left ventricular hypertrophy, and high–normal clinic systolic and/or diastolic blood pressure are predictors of MUCH and models including these variables appear to provide good diagnostic accuracy.^12^ Overall, the predictors found in these studies are, except pulse pressure, different to ours. Differences in study populations, study design, and evaluated variables, may explain these differences. Predictors needed to reveal MUCH in the high cardiovascular-risk patients following a recent MI may also be different, as compared to patients at lower cardiovascular risk, that have other cardiovascular properties and clinical characteristics.

#### Prediction and predictors of uncontrolled hypertension

We also studied the prediction of uncontrolled hypertension (i.e. including both MUCH and sustained uncontrolled hypertension) and obtained a slightly lower mean cross-validation AUC (0.76) for the best model, compared to prediction of MUCH, which still represents an acceptable discriminative performance.^22^

As expected, there was an overlap in important predictors such as discharge diagnoses of diabetes and hypertension, and pulse pressure at the first follow-up, for MUCH and uncontrolled hypertension. This is likely because most patients with uncontrolled hypertension (17 of 32) also had MUCH. Systolic blood pressure at the first follow-up was, as expected, an important predictor of uncontrolled hypertension. Interestingly, the level of physical activity at first follow-up emerged as a prominent predictor of uncontrolled hypertension, aligning with previous research that links cardiac rehabilitation and exercise with improved risk factor control and better cardiovascular outcomes.^33^

#### Clinical implications of identifying MUCH

Since masked hypertension/MUCH is associated with the same negative prognosis as sustained hypertension,^8,10^ it is appealing to identify these patients to improve their antihypertensive treatment. While current evidence support the benefit of out-of-office blood pressure measurements to guide antihypertensive treatment, the evidence for effects on morbidity and mortality in masked hypertension/MUCH is primarily circumstantial.^34^ Ongoing well-designed randomized controlled trials are expected to provide more definite results.^35^ Furthermore, the currently established definition for masked hypertension/MUCH is using an office blood pressure threshold of < 140/90 mm Hg,^4,7^ which is higher than the current treatment target recommendations.^3,4^ Using lower thresholds and targets may reveal different results.

#### Limitations

The finding of masked hypertension/MUCH is best confirmed with a second set of out-of-office blood pressure measurements since the reproducibility is limited, which was not performed.^36^ In addition, the small sample size of the current study may limit the machine learning-based evaluation of predictors. And although repeated cross-validation normally gives good estimates of the generalisability of prediction models, the models need to be evaluated through external validation. Furthermore, we do not have information on adherence to prescribed medications.

In conclusion, our study demonstrates that the clinically challenging blood pressure phenotype MUCH is common following a recent hospitalization for MI. Importantly, MUCH can be predicted using the readily available clinical variables diabetes, hypertension, and kidney dysfunction (eGFR). With appropriate validation, these predictors could be integrated into a machine learning model and implemented as a clinical decision support tool to improve the identification of MUCH, which may contribute to improved clinical outcomes in this high-risk population.

## Lead author biography


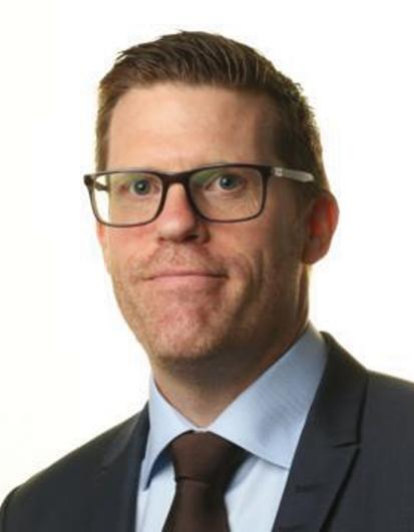


Dr Henrik Hellqvist is a specialist in internal medicine and cardiology, working at the Department of Cardiology, Danderyd University Hospital, Stockholm, Sweden. His clinical focus includes ischaemic heart disease, vascular medicine and cardiovascular risk. He is currently conducting research on assessment of arterial stiffness and vascular ageing for cardiovascular applications. Dr Hellqvist has a strong interest in emerging medical technologies and machine learning/artificial intelligence, and their applications in healthcare.

## Supplementary Material

oeaf138_Supplementary_Data

## Data Availability

The data underlying this article will be shared on reasonable request to the corresponding author, provided it is compatible with prevailing ethical approval and informed consent.

## References

[1] Visseren FLJ, Mach F, Smulders YM, Carballo D, Koskinas KC, Bäck M, Benetos A, Biffi A, Boavida J-M, Capodanno D, Cosyns B, Crawford C, Davos CH, Desormais I, Di Angelantonio E, Franco OH, Halvorsen S, Hobbs FDR, Hollander M, Jankowska EA, Michal M, Sacco S, Sattar N, Tokgozoglu L, Tonstad S, Tsioufis KP, van Dis I, van Gelder IC, Wanner C, Williams B, De Backer G, Regitz-Zagrosek V, Aamodt AH, Abdelhamid M, Aboyans V, Albus C, Asteggiano R, Bäck M, Borger MA, Brotons C, Čelutkienė J, Cifkova R, Cikes M, Cosentino F, Dagres N, De Backer T, De Bacquer D, Delgado V, Den Ruijter H, Dendale P, Drexel H, Falk V, Fauchier L, Ference BA, Ferrières J, Ferrini M, Fisher M, Fliser D, Fras Z, Gaita D, Giampaoli S, Gielen S, Graham I, Jennings C, Jorgensen T, Kautzky-Willer A, Kavousi M, Koenig W, Konradi A, Kotecha D, Landmesser U, Lettino M, Lewis BS, Linhart A, Løchen M-L, Makrilakis K, Mancia G, Marques-Vidal P, McEvoy JW, McGreavy P, Merkely B, Neubeck L, Nielsen JC, Perk J, Petersen SE, Petronio AS, Piepoli M, Pogosova NG, Prescott EIB, Ray KK, Reiner Z, Richter DJ, Rydén L, Shlyakhto E, Sitges M, Sousa-Uva M, Sudano I, Tiberi M, Touyz RM, Ungar A, Verschuren WMM, Wiklund O, Wood D, Zamorano JL, Smulders YM, Carballo D, Koskinas KC, Bäck M, Benetos A, Biffi A, Boavida J-M, Capodanno D, Cosyns B, Crawford CA, Davos CH, Desormais I, Di Angelantonio E, Franco Duran OH, Halvorsen S, Richard Hobbs FD, Hollander M, Jankowska EA, Michal M, Sacco S, Sattar N, Tokgozoglu L, Tonstad S, Tsioufis KP, Dis Iv, van Gelder IC, Wanner C, Williams B. 2021 ESC guidelines on cardiovascular disease prevention in clinical practice. Eur Heart J 2021;42:3227–3337. 34458905 10.1093/eurheartj/ehab484

[2] Byrne RA, Rossello X, Coughlan JJ, Barbato E, Berry C, Chieffo A, Claeys MJ, Dan G-A, Dweck MR, Galbraith M, Gilard M, Hinterbuchner L, Jankowska EA, Jüni P, Kimura T, Kunadian V, Leosdottir M, Lorusso R, Pedretti RFE, Rigopoulos AG, Rubini Gimenez M, Thiele H, Vranckx P, Wassmann S, Wenger NK, Ibanez B, Halvorsen S, James S, Abdelhamid M, Aboyans V, Marsan NA, Antoniou S, Asteggiano R, Bäck M, Capodanno D, Casado-Arroyo R, Cassese S, Čelutkienė J, Cikes M, Collet J-P, Ducrocq G, Falk V, Fauchier L, Geisler T, Gorog DA, Holmvang L, Jaarsma T, Jones HW, Køber L, Koskinas KC, Kotecha D, Krychtiuk KA, Landmesser U, Lazaros G, Lewis BS, Lindahl B, Linhart A, Løchen M-L, Mamas MA, McEvoy JW, Mihaylova B, Mindham R, Mueller C, Neubeck L, Niebauer J, Nielsen JC, Niessner A, Paradies V, Pasquet AA, Petersen SE, Prescott E, Rakisheva A, Rocca B, Rosano GMC, Sade LE, Schiele F, Siller-Matula JM, Sticherling C, Storey RF, Thielmann M, Vrints C, Windecker S, Wiseth R, Witkowski A, El Amine Bouzid M, Hayrapetyan H, Metzler B, Lancellotti P, Bajrić M, Karamfiloff K, Mitsis A, Ostadal P, Sørensen R, Elwasify T, Marandi T, Ryödi E, Collet J-P, Chukhrukidze A, Mehilli J, Davlouros P, Becker D, Guðmundsdóttir IJ, Crowley J, Abramowitz Y, Indolfi C, Sakhov O, Elezi S, Beishenkulov M, Erglis A, Moussallem N, Benlamin H, Dobilienė O, Degrell P, Balbi MM, Grosu A, Lakhal Z, ten Berg J, Pejkov H, Angel K, Witkowski A, De Sousa Almeida M, Chioncel O, Bertelli L, Stojkovic S, Studenčan M, Radšel P, Ferreiro JL, Ravn-Fischer A, Räber L, Marjeh MYB, Hassine M, Yildirir A, Parkhomenko A, Banning AP, Prescott E, James S, Arbelo E, Baigent C, Borger MA, Buccheri S, Ibanez B, Køber L, Koskinas KC, McEvoy JW, Mihaylova B, Mindham R, Neubeck L, Nielsen JC, Pasquet AA, Rakisheva A, Rocca B, Rossello X, Vaartjes I, Vrints C, Witkowski A, Zeppenfeld K. 2023 ESC guidelines for the management of acute coronary syndromes. Eur Heart J 2023;44:3720–3826. 37622654 10.1093/eurheartj/ehad191

[3] Mancia G, Kreutz R, Brunstrom M, Burnier M, Grassi G, Januszewicz A, Muiesan ML, Tsioufis K, Agabiti-Rosei E, Algharably EAE, Azizi M, Benetos A, Borghi C, Hitij JB, Cifkova R, Coca A, Cornelissen V, Cruickshank JK, Cunha PG, Danser AHJ, Pinho RMd, Delles C, Dominiczak AF, Dorobantu M, Doumas M, Fernández-Alfonso MS, Halimi J-M, Járai Z, Jelaković B, Jordan J, Kuznetsova T, Laurent S, Lovic D, Lurbe E, Mahfoud F, Manolis A, Miglinas M, Narkiewicz K, Niiranen T, Palatini P, Parati G, Pathak A, Persu A, Polonia J, Redon J, Sarafidis P, Schmieder R, Spronck B, Stabouli S, Stergiou G, Taddei S, Thomopoulos C, Tomaszewski M, Van de Borne P, Wanner C, Weber T, Williams B, Zhang Z-Y, Kjeldsen SE. 2023 ESH guidelines for the management of arterial hypertension the task force for the management of arterial hypertension of the European society of hypertension: endorsed by the international society of hypertension (ISH) and the European renal association (ERA). J Hypertens 2023;41:1874–2071. 37345492 10.1097/HJH.0000000000003480

[4] McEvoy JW, McCarthy CP, Bruno RM, Brouwers S, Canavan MD, Ceconi C, Christodorescu RM, Daskalopoulou SS, Ferro CJ, Gerdts E, Hanssen H, Harris J, Lauder L, McManus RJ, Molloy GJ, Rahimi K, Regitz-Zagrosek V, Rossi GP, Sandset EC, Scheenaerts B, Staessen JA, Uchmanowicz I, Volterrani M, Touyz RM; ESC Scientific Document Group 2024 ESC guidelines for the management of elevated blood pressure and hypertension. Eur Heart J 2024;45:3912–4018. 39210715 10.1093/eurheartj/ehae178

[5] Rahimi K, Bidel Z, Nazarzadeh M, Copland E, Canoy D, Ramakrishnan R, Pinho-Gomes A-C, Woodward M, Adler A, Agodoa L, Algra A, Asselbergs FW, Beckett NS, Berge E, Black H, Brouwers FPJ, Brown M, Bulpitt CJ, Byington RP, Cushman WC, Cutler J, Devereaux RB, Dwyer J, Estacio R, Fagard R, Fox K, Fukui T, Gupta AK, Holman RR, Imai Y, Ishii M, Julius S, Kanno Y, Kjeldsen SE, Kostis J, Kuramoto K, Lanke J, Lewis E, Lewis JB, Lievre M, Lindholm LH, Lueders S, MacMahon S, Mancia G, Matsuzaki M, Mehlum MH, Nissen S, Ogawa H, Ogihara T, Ohkubo T, Palmer CR, Patel A, Pfeffer MA, Pitt B, Poulter NR, Rakugi H, Reboldi G, Reid C, Remuzzi G, Ruggenenti P, Saruta T, Schrader J, Schrier R, Sever P, Sleight P, Staessen JA, Suzuki H, Thijs L, Ueshima K, Umemoto S, van Gilst WH, Verdecchia P, Wachtell K, Whelton P, Wing L, Yui Y, Yusuf S, Zanchetti A, Zhang Z-Y, Anderson C, Baigent C, Brenner BM, Collins R, de Zeeuw D, Lubsen J, Malacco E, Neal B, Perkovic V, Rodgers A, Rothwell P, Salimi-Khorshidi G, Sundström J, Turnbull F, Viberti G, Wang J, Chalmers J, Teo KK, Pepine CJ, Davis BR. Pharmacological blood pressure lowering for primary and secondary prevention of cardiovascular disease across different levels of blood pressure: an individual participant-level data meta-analysis. Lancet 2021;397:1625–1636. 33933205 10.1016/S0140-6736(21)00590-0 PMC8102467

[6] Denolle T, Pellen C, Serandour AL, Lebreton S, Revault d'Allonnes F. Persistence of uncontrolled hypertension post-cardiac rehabilitation in stable coronary patients. J Hum Hypertens 2022;36:537–543. 33963270 10.1038/s41371-021-00544-1

[7] Stergiou GS, Palatini P, Parati G, O’Brien E, Januszewicz A, Lurbe E, Persu A, Mancia G, Kreutz R. 2021 European society of hypertension practice guidelines for office and out-of-office blood pressure measurement. J Hypertens 2021;39:1293–1302. 33710173 10.1097/HJH.0000000000002843

[8] Thakkar HV, Pope A, Anpalahan M. Masked hypertension: a systematic review. Heart Lung Circ 2020;29:102–111. 31477513 10.1016/j.hlc.2019.08.006

[9] Cacciolati C, Hanon O, Dufouil C, Alperovitch A, Tzourio C. Categories of hypertension in the elderly and their 1-year evolution. The Three-City Study. J Hypertens 2013;31:680–689. 23412428 10.1097/HJH.0b013e32835ee0ca

[10] Banegas JR, Ruilope LM, de la Sierra A, Vinyoles E, Gorostidi M, de la Cruz JJ, Ruiz-Hurtado G, Segura J, Rodríguez-Artalejo F, Williams B. Relationship between clinic and ambulatory blood-pressure measurements and mortality. N Engl J Med 2018;378:1509–1520. 29669232 10.1056/NEJMoa1712231

[11] Kushiro T, Kario K, Saito I, Teramukai S, Sato Y, Okuda Y, Shimada K. Increased cardiovascular risk of treated white coat and masked hypertension in patients with diabetes and chronic kidney disease: the HONEST study. Hypertens Res 2017;40:87–95. 27511054 10.1038/hr.2016.87 PMC5222992

[12] Coccina F, Borrelli P, Pierdomenico AM, Pizzicannella J, Guagnano MT, Cuccurullo C, Di Nicola M, Renda G, Trubiani O, Cipollone F, Pierdomenico SD. Prediction of masked uncontrolled hypertension detected by ambulatory blood pressure monitoring. Diagnostics (Basel) 2022;12:3156. 36553162 10.3390/diagnostics12123156 PMC9777728

[13] Kim HJ, Shin JH, Lee Y, Kim JH, Hwang SH, Kim WS, Park S, Rhee SJ, Lee EM, Ihm SH, Pyun WB, Shin J. Clinical features and predictors of masked uncontrolled hypertension from the Korean ambulatory blood pressure monitoring registry. Korean J Intern Med 2021;36:1102–1114. 34134467 10.3904/kjim.2020.650 PMC8435491

[14] Hung MH, Shih LC, Wang YC, Leu H-B, Huang P-H, Wu T-C, Lin S-J, Pan W-H, Chen J-W, Huang C-C. Prediction of masked hypertension and masked uncontrolled hypertension using machine learning. Front Cardiovasc Med 2021;8:778306. 34869691 10.3389/fcvm.2021.778306 PMC8639874

[15] Chaikijurajai T, Laffin LJ, Tang WHW. Artificial intelligence and hypertension: recent advances and future outlook. Am J Hypertens 2020;33:967–974. 32615586 10.1093/ajh/hpaa102 PMC7608522

[16] Parati G, Goncalves A, Soergel D, Bruno RM, Caiani EG, Gerdts E, Mahfoud F, Mantovani L, McManus RJ, Santalucia P, Kahan T. New perspectives for hypertension management: progress in methodological and technological developments. Eur J Prev Cardiol 2023;30:48–60. 36073370 10.1093/eurjpc/zwac203

[17] Jernberg T, Attebring MF, Hambraeus K, Ivert T, James S, Jeppsson A, Lagerqvist B, Lindahl B, Stenestrand U, Wallentin L. The Swedish web-system for enhancement and development of evidence-based care in heart disease evaluated according to recommended therapies (SWEDEHEART). Heart 2010;96:1617–1621. 20801780 10.1136/hrt.2010.198804

[18] Beretta L, Santaniello A. Nearest neighbor imputation algorithms: a critical evaluation. BMC Med Inform Decis Mak 2016;16 Suppl 3:74. 27454392 10.1186/s12911-016-0318-z PMC4959387

[19] Tibshirani R . Regression shrinkage and selection via the lasso. J R Stat Soc Ser B (Methodological) 1996;58:267–288.

[20] Kursa MB, Rudnicki WR. Feature selection with the boruta package. J Stat Softw 2010;36:1–13.

[21] Gorostidi M, Vinyoles E, Banegas JR, de la Sierra A. Prevalence of white-coat and masked hypertension in national and international registries. Hypertens Res 2015;38:1–7. 25319601 10.1038/hr.2014.149

[22] Mandrekar JN . Receiver operating characteristic curve in diagnostic test assessment. J Thorac Oncol 2010;5:1315–1316. 20736804 10.1097/JTO.0b013e3181ec173d

[23] Breiman L . Random forests. Mach Learn 2001;45:5–32.

[24] Franklin SS, Thijs L, Li Y, Hansen TW, Boggia J, Liu Y, Asayama K, Björklund-Bodegård K, Ohkubo T, Jeppesen J, Torp-Pedersen C, Dolan E, Kuznetsova T, Stolarz-Skrzypek K, Tikhonoff V, Malyutina S, Casiglia E, Nikitin Y, Lind L, Sandoya E, Kawecka-Jaszcz K, Filipovský J, Imai Y, Wang J, Ibsen H, O’Brien E, Staessen JA. Masked hypertension in diabetes mellitus: treatment implications for clinical practice. Hypertension 2013;61:964–971. 23478096 10.1161/HYPERTENSIONAHA.111.00289 PMC3631136

[25] Franklin SS, O'Brien E, Staessen JA. Masked hypertension: understanding its complexity. Eur Heart J 2017;38:1112–1118. 27836914 10.1093/eurheartj/ehw502

[26] Siddiqui M, Judd EK, Jaeger BC, Bhatt H, Dudenbostel T, Zhang B, Edwards LJ, Oparil S, Calhoun DA. Out-of-clinic sympathetic activity is increased in patients with masked uncontrolled hypertension. Hypertension 2019;73:132–141. 30571547 10.1161/HYPERTENSIONAHA.118.11818 PMC6309788

[27] Boutouyrie P, Chowienczyk P, Humphrey JD, Mitchell GF. Arterial stiffness and cardiovascular risk in hypertension. Circ Res 2021;128:864–886. 33793325 10.1161/CIRCRESAHA.121.318061

[28] Briet M, Boutouyrie P, Laurent S, London GM. Arterial stiffness and pulse pressure in CKD and ESRD. Kidney Int 2012;82:388–400. 22534962 10.1038/ki.2012.131

[29] Antza C, Vazakidis P, Doundoulakis I, Bouras E, Haidich A, Stabouli S, Kotsis V. Masked and white coat hypertension, the double trouble of large arteries: a systematic review and meta-analysis. J Clin Hypertens (Greenwich) 2020;22:802–811. 32356941 10.1111/jch.13876 PMC8029862

[30] Rahman M, Wang X, Bundy JD, Charleston J, Cohen D, Cohen J, Drawz PE, Ghazi L, Horowitz E, Lash JP, Schrauben S, Weir MR, Xie D, Townsend RR. Prognostic significance of ambulatory BP monitoring in CKD: a report from the chronic renal insufficiency cohort (CRIC) study. J Am Soc Nephrol 2020;31:2609–2621. 32973085 10.1681/ASN.2020030236 PMC7608974

[31] Agarwal R, Pappas MK, Sinha AD. Masked uncontrolled hypertension in CKD. J Am Soc Nephrol 2016;27:924–932. 26163421 10.1681/ASN.2015030243 PMC4769206

[32] Agarwal R, Pappas MK. Delayed systolic blood pressure recovery following exercise as a mechanism of masked uncontrolled hypertension in chronic kidney disease. Nephrol Dial Transplant 2017;32:1710–1717. 27422961 10.1093/ndt/gfw266

[33] Xing Y, Yang SD, Wang MM, Feng Y-S, Dong F, Zhang F. The beneficial role of exercise training for myocardial infarction treatment in elderly. Front Physiol 2020;11:270. 32390856 10.3389/fphys.2020.00270 PMC7194188

[34] Hoshide S, Yano Y, Kanegae H, Kario K. Effect of lowering home blood pressure on subclinical cardiovascular disease in masked uncontrolled hypertension. J Am Coll Cardiol 2018;71:2858–2859. 29903355 10.1016/j.jacc.2018.04.017

[35] Parati G, Agabiti-Rosei E, Bakris GL, Bilo G, Branzi G, Cecchi F, Chrostowska M, De la Sierra A, Domenech M, Dorobantu M, Faria T, Huo Y, Jelaković B, Kahan T, Konradi A, Laurent S, Li N, Madan K, Mancia G, McManus RJ, Modesti PA, Ochoa JE, Octavio JA, Omboni S, Palatini P, Park JB, Pellegrini D, Perl S, Podoleanu C, Pucci G, Redon J, Renna N, Rhee MY, Rodilla Sala E, Sanchez R, Schmieder R, Soranna D, Stergiou G, Stojanovic M, Tsioufis K, Valsecchi MG, Veglio F, Waisman GD, Wang JG, Wijnmaalen P, Zambon A, Zanchetti A, Zhang Y. MASked-unconTrolled hypERtension management based on office BP or on ambulatory blood pressure measurement (MASTER) study: a randomised controlled trial protocol. BMJ Open 2018;8:e021038. 10.1136/bmjopen-2017-021038 PMC6303603 30573476

[36] Antza C, Farmakis I, Doundoulakis I, Akrivos E, Stalikas N, Zafeiropoulos S, Kostopoulos G, Stabouli S, Giannakoulas G, Kotsis V. Reproducibility of masked hypertension and office-based hypertension: a systematic review and meta-analysis. J Hypertens 2022;40:1053–1059. 35703872 10.1097/HJH.0000000000003111

