# Supplementary material for: Prevalence and prediction of masked uncontrolled hypertension in patients recently hospitalized for myocardial infarction
Source: Eur Heart J Open. 2025 Oct 14;5(6):oeaf138. doi: 10.1093/ehjopen/oeaf138 (PMC12603615; doi:10.1093/ehjopen/oeaf138)
Supplement: oeaf138_Supplementary_Data [file oeaf138_supplementary_data.docx]

**Supplementary information**This file contains the supplementary Tables S1-S4 and Figure S1.

Table S1: Variables assessed in machine learning

| No. | Situation | Variable | Category | Scale | Source | Tag |
| --- | --- | --- | --- | --- | --- | --- |
| 1 | In | Age, years | Demographics | Continuous | SH |  |
| 2 | In | Male sex | Demographics | Binary | SH |  |
| 3 | In | Height, cm | Anthropometry | Continuous | SH | Im |
| 4 | In | Weight, kg | Anthropometry | Continuous | SH | Im |
| 5 | In | BMI, kg/m^2^ | Anthropometry | Continuous | SH | Im |
| 6 | In | ECG rhythm in | Clinical assessment | Categorical | SH |  |
| 7 | In | ECG QRS in | Clinical assessment | Categorical | SH | Im |
| 8 | In | ECG ST-T in | Clinical assessment | Categorical | SH |  |
| 9 | In | HR in, bpm | Clinical assessment | Continuous | SH |  |
| 10 | In | SBP in, mmHg | Clinical assessment | Continuous | SH |  |
| 11 | In | DBP in, mmHg | Clinical assessment | Continuous | SH | Im |
| 12 | In | PP in, mm Hg | Clinical assessment | Continuous | SH-der. | Im |
| 13 | In | Pulmonary rales in | Clinical assessment | Binary | SH | Rv |
| 14 | In | Cardiogenic shock in | Clinical assessment | Binary | SH | Rv |
| 15 | In | Smoking history | Health condition | Categorical | SH |  |
| 16 | In | Snuff use history | Health condition | Categorical | SH |  |
| 17 | In | Previous MI | Health condition | Binary | SH |  |
| 18 | In | Previous PCI | Health condition | Binary | SH | Rv |
| 19 | In | Previous heart surgery | Health condition | Categorical | SH | Rv |
| 20 | In | Previous stroke | Health condition | Binary | SH | Rv |
| 21 | In | Occupation | History | Categorical | SH |  |
| 22 | In | Admission symptom/reason | History | Categorical | SH | Rv |
| 23 | In | ACEi, ARB in | Medication | Binary | SH |  |
| 24 | In | Anticoagulant in | Medication | Binary | SH | Rv |
| 25 | In | ASA in | Medication | Binary | SH |  |
| 26 | In | Other trc. inhibitor in | Medication | Binary | SH | Rv |
| 27 | In | Betablocker in | Medication | Binary | SH |  |
| 28 | In | CCB in | Medication | Binary | SH |  |
| 29 | In | Diabetes, insulin in | Medication | Binary | SH | Rv |
| 30 | In | Diabetes, po med. in | Medication | Binary | SH |  |
| 31 | In | Digoxin in | Medication | Binary | SH | Rv |
| 32 | In | Diuretics in | Medication | Binary | SH |  |
| 33 | In | MRA in | Medication | Binary | SH | Rv |
| 34 | In | Statin in | Medication | Binary | SH |  |
| 35 | In | Ezetimibe in | Medication | Binary | SH | Rv |
| 36 | In | Fibrate in | Medication | Binary | SH | Rv |
| 37 | In | PCSK9i in | Medication | Binary | SH | Rv |
| 38 | In | Long-acting nitrate in | Medication | Binary | SH | Rv |
| 39 | In | Hyperlipidaemia, med. in | Medication | Binary | SH |  |
| 40 | Care | LVEF, % | Clinical assessment | Categorical | SH | Rm |
| 41 | Care | Max troponin T, mmol/l | Blood tests | Continuous | SH | Im |
| 42 | Care | Cholesterol, mmol/l | Blood tests | Continuous | SH | Im |
| 43 | Care | TG, mmol/l | Blood tests | Continuous | SH | Im |
| 44 | Care | HDL, mmol/l | Blood tests | Continuous | SH | Im |
| 45 | Care | LDL, mmol/l | Blood tests | Continuous | SH | Im |
| 46 | Care | LDL/HDL ratio | Blood tests | Continuous | SH | Im |
| 47 | Care | Glucose, mmol/l | Blood tests | Continuous | SH | Im |
| 48 | Care | HbA1c, mmol/l | Blood tests | Continuous | SH | Rm |
| 49 | Care | eGFR, ml/min/1.73m^2^ | Blood tests | Continuous | SH-der. |  |
| 50 | Care | CRP, mmol/l | Blood tests | Continuous | SH | Rm |
| 51 | Care | Hb, mmol/l | Blood tests | Continuous | SH | Im |
| 52 | Care | AF discovered at care | Health condition | Binary | SH |  |
| 53 | Care | HF at care | Health condition | Binary | SH | Im |
| 54 | Care | PCI performed | Health condition | Binary | SH | Rv |
| 55 | Care | CABG performed | Health condition | Binary | SH | Rv |
| 56 | Out | ECG rhythm out | Clinical assessment | Categorical | SH | Rv |
| 57 | Out | MI type (NSTEMI/STEMI) | Health condition | Categorical | SH |  |
| 58 | Out | AF/AFL diagnosis | Health condition | Binary | SH-der. |  |
| 59 | Out | COPD diagnosis | Health condition | Binary | SH-der. | Rv |
| 60 | Out | Hyperlipidaemia diagnosis | Health condition | Binary | SH-der. |  |
| 61 | Out | OSA diagnosis | Health condition | Binary | SH-der. | Rv |
| 62 | Out | Hypertension diagnosis | Health condition | Binary | SH-der. |  |
| 63 | Out | Heart failure diagnosis | Health condition | Binary | SH-der. | Im |
| 64 | Out | Diabetes diagnosis | Health condition | Binary | SH-der. |  |
| 65 | Out | Kidney failure diagnosis | Health condition | Binary | SH-der. | Rv |
| 66 | Out | Impaired OGTT diagnosis | Health condition | Binary | SH-der. |  |
| 67 | Out | ACEi, ARB out | Medication | Binary | SH |  |
| 68 | Out | Anticoagulant out | Medication | Binary | SH |  |
| 69 | Out | ASA out | Medication | Binary | SH | Rv |
| 70 | Out | Other trc. inhibitor out | Medication | Binary | SH | Rv |
| 71 | Out | Betablocker out | Medication | Binary | SH |  |
| 72 | Out | CCB out | Medication | Binary | SH |  |
| 73 | Out | Diabetes, insulin out | Medication | Binary | SH | Rv |
| 74 | Out | Diabetes, po med. out | Medication | Binary | SH |  |
| 75 | Out | Digoxin out | Medication | Binary | SH | Rv |
| 76 | Out | Diuretics out | Medication | Binary | SH |  |
| 77 | Out | MRA out | Medication | Binary | SH |  |
| 78 | Out | Statin out | Medication | Binary | SH | Rv |
| 79 | Out | Ezetimibe out | Medication | Binary | SH | Rv |
| 80 | Out | Fibrate out | Medication | Binary | SH | Rv |
| 81 | Out | PCSK9i out | Medication | Binary | SH | Rv |
| 82 | Out | Long-acting nitrate out | Medication | Binary | SH | Rv |
| 83 | Out | BP-lowering medication count | Medication | Continuous | SH-der. |  |
| 84 | FU1 | SBP FU1, mm Hg | Clinical assessment | Continuous | SH | Im |
| 85 | FU1 | DBP FU1, mm Hg | Clinical assessment | Continuous | SH | Im |
| 86 | FU1 | PP FU1, mm Hg | Clinical assessment | Continuous | SH-der. | Im |
| 87 | FU1 | SBP FU1 130–139 mm Hg | Clinical assessment | Binary | SH-der. | Im |
| 88 | FU1 | DBP FU1 85-89 mm Hg | Clinical assessment | Binary | SH-der. | Im |
| 89 | FU1 | Waist circumference, cm | Clinical assessment | Continuous | SH | Rm |
| 90 | FU1 | Stopped smoking | Health condition | Binary | SH | Rm |
| 91 | FU1 | Stopped using snuff | Health condition | Binary | SH | Rm |
| 92 | FU1 | Physical activity FU1, level | Health condition | Continuous | SH | Im |
| 93 | FU1 | Participated in heart school | History | Binary | SH | Im |
| 94 | FU1 | Participated in cardiac rehabilitation training | History | Binary | SH | Im |
| 95 | FU1 | BP-lowering medication count FU1 | Medication | Continuous | SH-der. | Im |
| 96 | FU1 | ACEi, ARB FU1 | Medication | Binary | SH | Im |
| 97 | FU1 | Betablocker FU1 | Medication | Binary | SH | Im |
| 98 | FU1 | CCB FU1 | Medication | Binary | SH | Im |
| 99 | FU1 | Diuretics FU1 | Medication | Binary | SH | Rv |
| 100 | FU1 | MRA FU1 | Medication | Binary | SH | Rv |
| 101 | FU1 | Long-acting nitrate FU1 | Medication | Binary | SH | Rv |
| 102 | FU1 | Triglycerides FU1 | Blood tests | Continuous | SH | Rm |
| 103 | FU1 | Cholesterol FU1 | Blood tests | Continuous | SH | Rm |
| 104 | FU1 | HDL FU1 | Blood tests | Continuous | SH | Rm |
| 105 | FU1 | LDL FU1, mmol/l | Blood tests | Continuous | SH | Rm |
| 106 | FU1 | LDL/HDL ratio FU1 | Blood tests | Continuous | SH | Rm |

Clinical variables from the SWEDEHEART (Swedish Web-system for Enhancement and Development of Evidence-based care in Heart disease Evaluated According to Recommended Therapies) registry that were selected for machine learning. In pre-processing variables with tag “Rm” were removed due missing values and those with tag “Rv” due to low variance, and variables with tag “Im” had imputed values. SH, SWEDEHEART; SH-der., SH-derived; BMI, body mass index; HR, heart rate; SBP, systolic blood pressure; DBP, diastolic blood pressure; PP, pulse pressure; MI; myocardial infarction; PCI, percutaneous coronary intervention; CABG, coronary artery bypass graft; STEMI, ST-elevation MI; NSTEMI, non-ST-elevation MI, ACEi, angiotensin-converting enzyme inhibitor; ARB, angiotensin receptor antagonist; ASA, acetylsalicylic acid; CCB, calcium channel blocker; PCSK9i, proprotein convertase subtilisin/kexin type 9 inhibitor; LVEF, left ventricular ejection fraction; AF, atrial fibrillation; HF, heart failure; COPD, chronic obstructive pulmonary disease; OSA, obstructive sleep apnea; OGTT, oral glucose tolerance test; FU1, first follow-up.

**Table S2: ABPM metrics according to 24-h blood pressure phenotype post-myocardial infarction**

| Characteristic | All | Normotension | White coat hypertension | Masked hypertension | Sustained hypertension | *P* | *Q* |
| --- | --- | --- | --- | --- | --- | --- | --- |
| n | 99 | 59 | 5 | 17 | 15 |  |  |
| 24-h SBP, mmHg | 124 ± 13 | 116 ± 9 | 120 ± 7 | 136 ± 7 | 141 ± 7 | <0.001 | <0.001 |
| 24-h DBP, mmHg | 72 ± 7 | 69 ± 4 | 69 ± 3 | 76 ± 7 | 80 ± 8 | <0.001 | <0.001 |
| 24-h SBP SD, mm Hg | 14 ± 4 | 14 ± 4 | 17 ± 4 | 15 ± 3 | 16 ± 4 | 0.008 | 0.16 |
| 24-h DBP SD, mm Hg | 11 ± 3 | 10 ± 2 | 13 ± 3 | 11 ± 2 | 12 ± 3 | 0.018 | 0.36 |
| 24-h HR, bpm | 66 ± 9 | 65 ± 9 | 64 ± 8 | 68 ± 10 | 68 ± 11 | 0.54 | >0.99 |
| Day SBP, mmHg | 130 ± 14 | 121 ± 10 | 130 ± 6 | 141 ± 7 | 149 ± 7 | <0.001 | <0.001 |
| Day DBP, mmHg | 77 ± 8 | 74 ± 6 | 76 ± 1 | 79 ± 7 | 85 ± 9 | <0.001 | <0.001 |
| Day SBP SD, mm Hg | 13 ± 4 | 12 ± 3 | 13 ± 4 | 14 ± 3 | 13 ± 4 | 0.14 | >0.99 |
| Day DBP SD, mm Hg | 9 ± 3 | 8 ± 3 | 10 ± 3 | 10 ± 2 | 10 ± 3 | 0.065 | >0.99 |
| Day HR, bpm | 70 ± 11 | 69 ± 10 | 70 ± 9 | 72 ± 11 | 72 ± 15 | 0.65 | >0.99 |
| Night SBP, mmHg | 115 ± 14 | 108 ± 10 | 106 ± 9 | 127 ± 11 | 130 ± 8 | <0.001 | <0.001 |
| Night DBP, mmHg | 65 ± 7 | 63 ± 6 | 60 ± 5 | 69 ± 6 | 72 ± 8 | <0.001 | <0.001 |
| Night SBP SD, mm Hg | 10 ± 3 | 10 ± 3 | 10 ± 3 | 11 ± 3 | 11 ± 3 | 0.22 | >0.99 |
| Night DBP SD, mm Hg | 8 ± 2 | 7 ± 2 | 9 ± 2 | 9 ± 3 | 9 ± 2 | 0.002 | 0.035 |
| Night HR, bpm | 61 ± 10 | 60 ± 9 | 56 ± 9 | 63 ± 10 | 64 ± 10 | 0.28 | >0.99 |
| Systolic dipping, % | 11 ± 8 | 11 ± 8 | 18 ± 7 | 10 ± 6 | 12 ± 5 | 0.18 | >0.99 |
| Diastolic dipping, % | 15 ± 8 | 15 ± 9 | 21 ± 7 | 13 ± 4 | 15 ± 8 | 0.32 | >0.99 |
| ABPM, 24-h hypertension | 32 (33%) | 0 (0%) | 0 (0%) | 17 (100%) | 15 (100%) | <0.001 | <0.001 |
| ABPM, daytime hypertension | 39 (40%) | 4 (6.8%) | 2 (40%) | 17 (100%) | 15 (100%) | <0.001 | <0.001 |
| ABPM, night-time hypertension | 39 (41%) | 10 (17%) | 0 (0%) | 16 (94%) | 13 (87%) | <0.001 | <0.001 |

Values presented as mean values ± SD, median values, or n (%), with *P* level of significance between-groups and *Q*, showing adjusted *P* values after correction for multiple testing. ABPM, ambulatory blood pressure monitoring; SBP, systolic blood pressure; DBP, diastolic blood pressure; HR, heart rate.

**Table S3: Top predictors of MUCH and sustained uncontrolled hypertension according to 24-h blood pressure phenotype post-myocardial infarction**

| Characteristic | All | Normotension | White coat hypertension | Masked hypertension | Sustained hypertension | *P* | *Q* |
| --- | --- | --- | --- | --- | --- | --- | --- |
| n | 99 | 59 | 5 | 17 | 15 |  |  |
| Age, years | 62.1 ± 8.2 | 60.8 ± 7.7 | 64.6 ± 12.5 | 65.5 ± 9.2 | 62.4 ± 7.7 | 0.046 | 0.41 |
| Diabetes diagnosis | 19 (20%) | 7 (12%) | 0 (0%) | 7 (41%) | 3 (21%) | 0.038 | 0.34 |
| Hypertension diagnosis | 58 (60%) | 28 (48%) | 4 (80%) | 15 (88%) | 9 (64%) | 0.014 | 0.13 |
| MI type (NSTEMI/STEMI) |  |  |  |  |  | 0.34 | >0.99 |
| NSTEMI | 50 (52%) | 27 (47%) | 2 (40%) | 12 (71%) | 7 (50%) |  |  |
| STEMI | 47 (48%) | 31 (53%) | 3 (60%) | 5 (29%) | 7 (50%) |  |  |
| eGFR, ml/min/1.73m^2^ | 89 ± 15 | 90 ± 15 | 95 ± 10 | 80 ± 14 | 94 ± 15 | 0.034 | 0.31 |
| Cholesterol, mmol/l | 5.1 ± 1.2 | 5.1 ± 1.3 | 4.5 ± 0.9 | 5.1 ± 1.3 | 5.5 ± 0.9 | 0.48 | >0.99 |
| SBP FU1, mm Hg | 130 ± 16 | 125 ± 14 | 136 ± 6 | 136 ± 15 | 135 ± 13 | 0.008 | 0.075 |
| PP FU1, mm Hg | 53 ± 14 | 49 ± 10 | 55 ± 9 | 62 ± 19 | 56 ± 14 | 0.027 | 0.25 |
| Physical activity FU1, level | 5.3 ± 1.6 | 5.5 ± 1.6 | 6.5 ± 0.6 | 4.9 ± 1.5 | 4.8 ± 1.7 | 0.053 | 0.48 |

Values presented as mean values ± SD, median values, or n (%), with *P* level of significance between-groups and *Q*, showing adjusted *P* values after correction for multiple testing. ^1^ denotes predictors among the top five selected by LASSO or Boruta, respectively, in prediction of masked uncontrolled hypertension (MUCH), and ^2^ denotes the top predictors for uncontrolled hypertension (MUCH and sustained uncontrolled hypertension), using the same selection approach. MI, myocardial infarction; STEMI, ST-elevation MI; NSTEMI, non-ST-elevation MI; eGFR, estimated glomerular filtration rate; SBP, systolic blood pressure; PP, pulse pressure; FU1, first follow-up.

Table S4: Prediction of uncontrolled hypertension post-myocardial infarction

| Algorithm | Predictors | Mean CV AUC | Sensitivity | Specificity | PPV | NPV | Accuracy |
| --- | --- | --- | --- | --- | --- | --- | --- |
| Random forest | Diabetes; SBP at FU1; PP at FU1; Phys. act. at FU1, level | 0.76 | 0.87 | 0.63 | 0.54 | 0.91 | 0.71 |
| Random forest | Diabetes; SBP at FU1; PP at FU1; Hypertension; Phys. act. at FU1, level | 0.76 | 0.81 | 0.73 | 0.60 | 0.88 | 0.76 |
| Random forest | Diabetes; SBP at FU1; Hypertension; Phys. act. at FU1, level | 0.76 | 0.65 | 0.78 | 0.59 | 0.82 | 0.73 |
| Random forest | SBP at FU1; PP at FU1; Phys. act. at FU1, level | 0.76 | 0.81 | 0.73 | 0.60 | 0.88 | 0.76 |
| Random forest | Diabetes; PP at FU1; Hypertension; Phys. act. at FU1, level | 0.75 | 0.84 | 0.63 | 0.53 | 0.89 | 0.70 |
| Logistic regression | Diabetes; SBP at FU1; Phys. act. at FU1, level | 0.73 | 0.65 | 0.73 | 0.54 | 0.81 | 0.70 |
| LASSO | Diabetes; SBP at FU1; Phys. act. at FU1, level | 0.73 | 0.61 | 0.78 | 0.58 | 0.80 | 0.72 |
| Logistic regression | Diabetes; SBP at FU1 | 0.73 | 0.68 | 0.65 | 0.49 | 0.80 | 0.66 |
| LASSO | Diabetes; SBP at FU1 | 0.73 | 0.68 | 0.65 | 0.49 | 0.80 | 0.66 |
| Logistic regression | Diabetes; SBP at FU1; Hypertension; Phys. act. at FU1, level | 0.72 | 0.84 | 0.49 | 0.45 | 0.86 | 0.61 |
| LASSO | Diabetes; SBP at FU1; Hypertension; Phys. act. at FU1, level | 0.72 | 0.84 | 0.49 | 0.45 | 0.86 | 0.61 |
| Logistic regression | Diabetes; PP at FU1; Hypertension; Phys. act. at FU1, level | 0.72 | 0.55 | 0.87 | 0.68 | 0.80 | 0.77 |
| LASSO | Diabetes; PP at FU1; Hypertension; Phys. act. at FU1, level | 0.72 | 0.55 | 0.87 | 0.68 | 0.80 | 0.77 |
| Logistic regression | Diabetes; PP at FU1; Phys. act. at FU1, level | 0.72 | 0.68 | 0.76 | 0.58 | 0.83 | 0.73 |
| LASSO | Diabetes; PP at FU1; Phys. act. at FU1, level | 0.72 | 0.68 | 0.76 | 0.58 | 0.83 | 0.73 |

Prediction of uncontrolled hypertension using different machine learning algorithms in an exhaustive evaluation of all top predictors from variable importance analyses. The list is sorted on mean repeated cross-validation (CV) receiver operating characteristic area under the curve (AUC). The mean predicted probability for each observation and the threshold at maximum Youden’s index were used to calculate the performance metrics sensitivity, specificity, positive predictive value (PPV), negative predictive value (NPV) and accuracy. Diabetes and hypertension represent diagnoses at discharge. SBP; systolic blood pressure; PP, pulse pressure FU1, first follow-up; LASSO, least absolute shrinkage and selection operator.

**
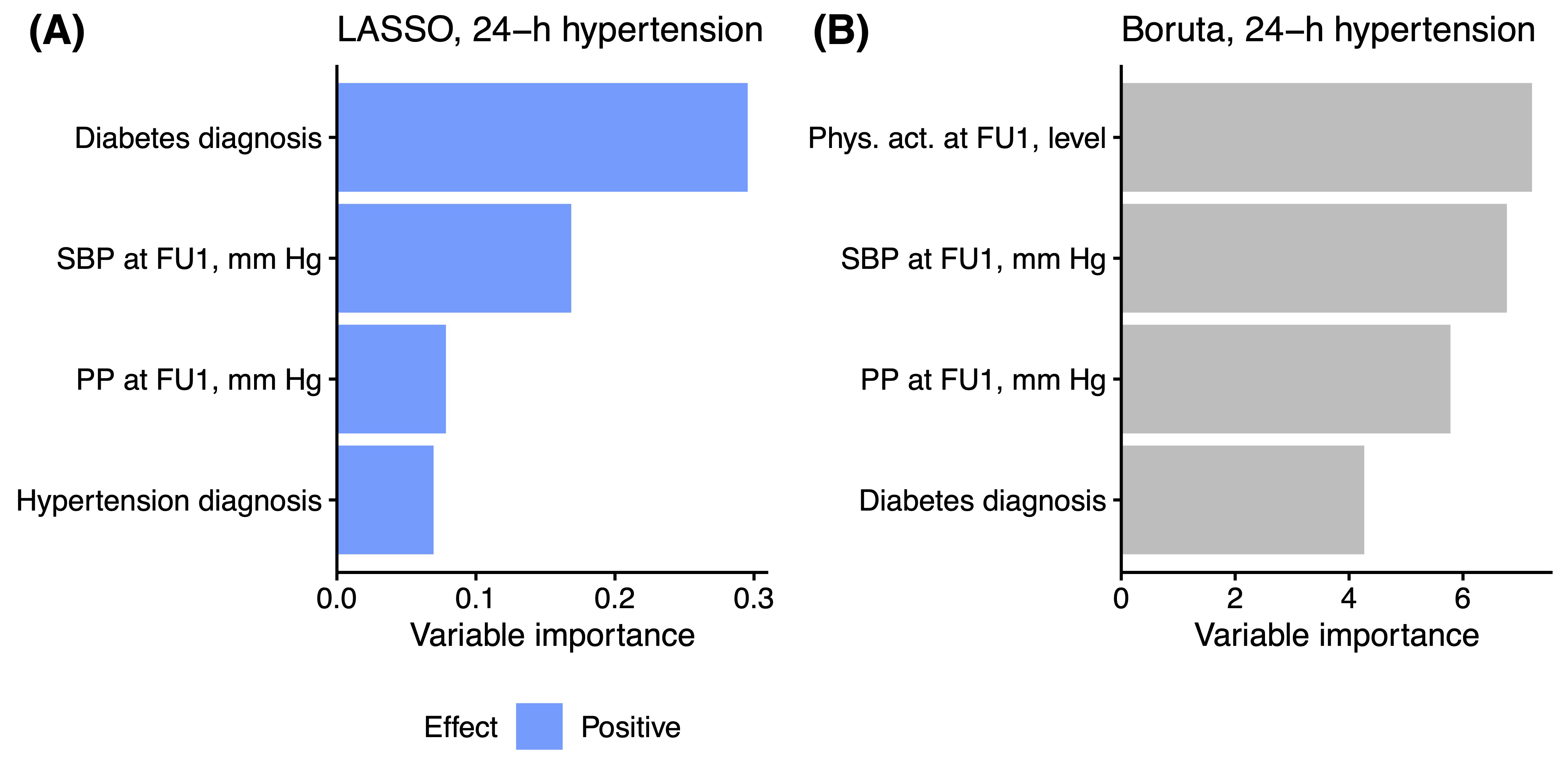
Figure S1.** Variable importance scores from machine learning in prediction of uncontrolled 24-h hypertension using (A) LASSO and (B) Boruta algorithms. The scores use relative scales, and LASSO also includes the effect direction. LASSO, least absolute shrinkage and selection operator; DM, diabetes mellitus; SBP, systolic blood pressure; PP, pulse pressure; FU1, first follow-up.
